# Diversity and composition of vaginal microbiota of pregnant women at risk for transmitting Group B Streptococcus treated with intrapartum penicillin

**DOI:** 10.1371/journal.pone.0169916

**Published:** 2017-02-08

**Authors:** Luiz Fernando Wurdig Roesch, Rita C. Silveira, Andréa L. Corso, Priscila Thiago Dobbler, Volker Mai, Bruna S. Rojas, Álvaro M. Laureano, Renato S. Procianoy

**Affiliations:** 1 Centro Interdisciplinar de Pesquisas em Biotecnologia – CIP-Biotec, Campus São Gabriel, Universidade Federal do Pampa, São Gabriel, Rio Grande do Sul, Brasil; 2 Unidade de Neonatologia do Hospital de Clínicas de Porto Alegre, Universidade Federal do Rio Grande do Sul, Porto Alegre, Rio Grande do Sul, Brasil; 3 Department of Epidemiology, College of Public Health and Health Professions and College of Medicine, Emerging Pathogens Institute, University of Florida, Gainesville, Florida, United States of America; Fred Hutchinson Cancer Research Center, UNITED STATES

## Abstract

**Background:**

Administering intravenous antibiotics during labor to women at risk for transmitting Group B Streptococcus (GBS) can prevent infections in newborns. However, the impact of intrapartum antibiotic prophylaxis on mothers’ microbial community composition is largely unknown. We compared vaginal microbial composition in pregnant women experiencing preterm birth at ≤ 32 weeks gestation that received intrapartum antibiotic prophylaxis with that in controls.

**Methods:**

Microbiota in vaginal swabs collected shortly before delivery from GBS positive women that received penicillin intravenously during labor or after premature rupture of membranes was compared to controls. Microbiota was analyzed by 16S rRNA sequencing using the PGM Ion Torrent to determine the effects of penicillin use during hospitalization and GBS status on its composition.

**Results:**

Penicillin administration was associated with an altered vaginal microbial community composition characterized by increased microbial diversity. *Lactobacillus* sp. contributed only 13.1% of the total community in the women that received penicillin compared to 88.1% in the controls. *Streptococcus* sp. were present in higher abundance in GBS positive woman compared to controls, with 60% of the total vaginal microbiota in severe cases identified as *Streptococcus sp*.

**Conclusions:**

Vaginal communities of healthy pregnant women were dominated by *Lactobacillu*s sp. and contained low diversity, while Group B *Streptococcus* positive women receiving intrapartum antibiotic prophylaxis had a modified vaginal microbiota composition with low abundance of *Lactobacillus* but higher microbial diversity.

## Introduction

The ability to detect microbes in various environments has been tremendously improved by the availability of high throughout sequencing approaches, significantly increasing our appreciation for the importance of microbes to contribute to human health. The human being is now understood as a superorganism [1] governed by a single human genome and by multiple microbial genomes inhabiting several body sites. While many clinical disorders appear to correlate with alterations in microbial communities evidence for causal associations is sparse. The normal human vaginal microbiota seems to contribute to maintaining microbial balance, which appears to protect from microbial diseases including bacterial vaginosis [2,3] and urinary tract infections [4]. The vaginal microbiomes vary significantly among women, especially in those of different ethnicities [5]. During pregnancy microbiota become less diverse and enriched in *Lactobacillus* sp. [6–8]. Unusual changes in vaginal microbiome including the appearance of opportunistic pathogens during pregnancy, potentially induced by administration of prepartum antibiotics, might increase risk for diseases with serious implications to the mother and their neonate. However, to date little is known about the effects of prepartum antibiotics on vaginal microbiota in pregnant women, especially those delivering prematurely at gestational age <32 weeks.

Group B Streptococcus (GBS) has been recognized as the leading infectious cause of early onset sepsis in the United States of America (USA) in the early 1970, remaining to date the main cause of sepsis of maternal origin in that country. Maternal colonization with GBS in genitourinary or gastrointestinal tracts is the primary risk factor for disease especially in vaginally delivered infants [9]. Clinical trials and observational studies demonstrated that administering intravenous antibiotics during labor to women at risk for transmitting GBS to their newborn can prevent invasive disease in the first week of life. Guidelines for the prevention of early-onset GBS disease recommend culture-based screening of pregnant women at 35–37 weeks of gestational age to identify women that should receive intrapartum antibiotic prophylaxis [9].

Preterm delivery is an important risk factor for early-onset GBS sepsis with a higher mortality compared to full-term infants. The Center for Disease Control and Prevention (CDC) recommends antibiotic prophylaxis for all pregnant women that tested positive for GBS colonization (except in the instance of cesarean delivery performed before onset of labor on a woman with intact amniotic membranes) and for women with preterm delivery with unknown GBS status [9].

While intrapartum antibiotic prophylaxis can prevent neonatal sepsis, the mothers’ microbial composition is impacted with potential effects on the vaginal microbial milieu during delivery and having possible implications on microbiota transmission to the baby. Here we aimed to analyze vaginal microbial composition in pregnant women at risk for transmitting GBS receiving intrapartum antibiotic prophylaxis and compared to control groups using cultivation-independent 16S rRNA sequencing technology.

## Materials and methods

### Experiment design and sampling strategy

Patients were recruited from the Neonatology Section of Hospital de Clínicas de Porto Alegre (HCPA), Brazil. Expecting mothers were enrolled at hospital admission for their delivery and provided written informed consent. The study protocol was approved by the ethics committee of Hospital de Clínicas de Porto Alegre (HCPA). Mothers delivering at gestational age ≤ 32 weeks were included in the study. The exclusion criteria were: 1) mother with HIV and congenital infections, 2) drug user or alcoholic and 3) fetus with congenital malformations. The study used a convenience sampling strategy. From a total of 114 women screened, nine patients were GBS positive and received intrapartum penicillin. Nine patients were GBS negative and did not receive penicillin. An additional control group of nine patients with unknown pre-delivery GBS status was included. Six of them received penicillin but were GBS negative and three patients were GBS positive but didn’t receive penicillin due to elective cesarean delivery with intact amniotic membranes. Penicillin was intravenously administered initially at a dose of 5.000.000 IU and additional doses of 2.500.000U-3.000.000 IU were administered every 4 hours until delivery according to CDC protocol [9].

Maternal variables that we collected included: maternal age, type of delivery, occurrence of preeclampsia and maternal gestational diabetes, presence of histologic or clinical chorioamnionitis (maternal fever, uterine hypertonicity, purulent or foul smelling amniotic fluid, maternal leukocytosis or fetal tachycardia) or urinary tract infection (positive urine culture). Antenatal steroid use was defined as the completion of 2 doses of betamethasone 24 hours apart or 4 doses of dexamethasone given 12 hours apart. Diagnosis of maternal preeclampsia was based on arterial hypertension (blood pressure ≥140 mmHg systolic and/or ≥90 mm Hg diastolic) developing after 20 weeks of gestation and proteinuria >300 mg in a 24-hour urine sample in the absence of previous hypertension or renal disease [10].

Vaginal swabs (Sterile Specimen Collection Swabs to collect specimens from soft tissue surfaces–*Labor swab*^®^) were collected before delivery (at least four hours after receiving the first penicillin dose) through vaginal introitus by rotating five times a sterilized swab along de lumen with a circular motion. Speculum was not used. Immediately after swabbing, the sample was placed in a sterile tube and kept at -80°C for later analysis.

### Microbial DNA extraction, 16S library preparation and sequencing

Microbial DNA was isolated from vaginal swab using the QIAamp Fast DNA Stool Mini Kit (Qiagen, Valencia, CA, USA) following the manufacturer instructions. DNA quality was determined by spectrophotometry using NanoVue^™^ spectrophotometer (GE Healthcare, Chicago, IL, USA). All DNA samples were stored at -80°C until use. The V4 region of the 16S rRNA gene was amplified and sequenced using the PGM Ion Torrent (Thermo Fisher Scientific, Waltham, MA, USA) with the bacterial/archaeal primers 515F and 806R [11]. Multiple samples were PCR-amplified using barcoded primers linked with the Ion adapter “A” sequence (5′-CCATCTCATCCCTGCGTGTCTCCGACTCAG-3′) and Ion adapter “P1” sequence (5′-CCTCTCTATGGGCAGTCGGTGAT-3′) to obtain a sequence of primer composed for A-barcode-806R and P1-515F adapter and primers. Each of the 25 μL of PCR mixture consisted of 2U of Platinum^®^ Taq DNA High Fidelity Polymerase (Invitrogen, Carlsbad, CA, USA), 4 μL 10X High Fidelity PCR Buffer, 2 mM MgSO4, 0.2 mM dNTP’s, 0.1 μM of both the 806R barcoded primer and the 515F primer, 25μg of Ultrapure BSA (Invitrogen, Carlsbad, CA, USA) and approximately 50 ng of DNA template. PCR conditions used were: 95°C for 5 min, 35 cycles of 94°C per 45s denaturation; 56°C per 45s annealing and 72°C per 1 min extension; followed by 72°C per 10 min. The resulting PCR products were purified with the Agencourt^®^ AMPure^®^ XP Reagent (Beckman Coulter, Brea, CA, USA) and the final concentration of the PCR product was quantified by using the Qubit Fluorometer kit (Invitrogen, Carlsbad, CA, USA) following manufacturer's recommendations. Finally, the reactions were combined in equimolar concentrations to create a mixture composed by 16S gene amplified fragments of each sample. This composite sample was used for library preparation with Ion OneTouch^™^ 2 System with the Ion PGM^™^ Template OT2 400 Kit Template (Thermo Fisher Scientific, Waltham, MA, USA). The sequencing was performed using Ion PGM^™^ Sequencing 400 on Ion PGM^™^ System using Ion 318^™^ Chip v2 with a maximum of 30 samples per microchip. All relevant data are fully available without restriction. Raw sequences were deposited in the NCBI Sequence Read Archive under the BioProject ID PRJNA354838 accession numbers SAMN06053701 to SAMN6053727.

### Sequence processing and statistical analysis

The 16S rRNA raw sequences were analyzed following the recommendations of the Brazilian Microbiome Project [12]. Briefly, the OTU (Operational Taxonomic Unit) table was built using the UPARSE pipeline [13] in which the reads were truncated at 200 bp and quality filtered using a maximum expected error of 0.5. Filtered reads were dereplicated and singletons were removed. The sequences were clustered into OTUs at 97% similarity cutoff and chimeras checked to obtain representative sequences for each microbial phylotype. Taxonomic classification was carried-out in QIIME^(12)^ based on the UCLUST method against the Greengenes 13.5 database [14] with a confidence threshold of 80%. Sampling effort was estimated using Good’s coverage [15].

For the estimation of alpha diversity and richness, the data set was rarefied to the same number of sequences [16] and the Observed OTU richness and the Shannon diversity index were calculated and further plotted using the “phyloseq” package [17].

To test the hypothesis that antibiotic and/or GBS infection shape the vaginal microbial community the 16S rRNA gene dataset was rarefied to the same number of sequences^(16)^ and used to construct a dissimilarity matrix generated by Bray Curtis and Binary distances using the “phyloseq” package in R. The matrixes were ordinated by Principal Coordinate Analysis (PCoA) and *adonis* function was used to verify the strength and statistical significance of groups among treatments with the vegan package [18].

Analysis of Metagenomic Profiles v2 (STAMP) software package was used to determine differences in the relative abundances of categories (i.e. genus) between treatments. Differences among treatments were calculated using the ANOVA for multiple comparisons followed by the Tukey-Kramer *post hoc* test. Taxonomic unities with difference between proportions below 1% were excluded from the analysis. The clinical data were reported as mean ± SD or frequencies and percentages. The significance of differences between the groups of clinical data was evaluated using the t test. The Fisher exact test was used for analysis of dichotomous characteristics.

## Results

Despite the small number of women sampled in this study, we showed that the groups were similar regarding maternal age, gestational age, birth weight of newborn and maternal comorbidities such as gestational diabetes, choriamnionitis, pre-eclampsia and urinary tract infection Table 1. All pregnant mothers received corticosteroids, according to the protocol of the Unit.

**Table 1:** Maternal variables used for comparison between groups.

	Group 1*	Group 2**	Group 3***	Group 4****	*p-value*
	n = 9	n = 3	n = 6	n = 9	
Maternal age (years)^1^	26.2 ± 6.6	25.0 ± 7.0	24.5±4.6	25.7±8.7	0.97
Gestational age (weeks)^1^	29.8 ± 2.1	31.6 ± 1.4	31.4±2.1	31.1±1.7	0.29
Birthweight (g)^1^	1400.6 ±426.7	1399.2 ±509.0	1803.3 ±511.7	1793.6 ±455.0	0.24
Cesarean delivery^2^	8 (88.9%)	3 (100.0%)	0 (0%)	6 (66,7%)	0.01
Chorioamnionitis^2^	2 (22.2%)	1 (33.3%)	2 (33.3%)	3 (33.3%)	0.95
Antenatal steroids^2^	9 (100%)	3 (100%)	6 (100%)	9 (100%)	1.00
Preeclampsia^2^	5 (55.6%)	1 (33.3%)	0 (0%)	2 (22,2%)	0.12
Gestational diabetes^2^	1 (11.1%)	0 (0%)	2 (33.3%)	3 (33.3%)	0.54
Urinary tract infection^2^	0 (0%)	1 (33.3%)	0 (0%)	2 (22,2%)	0.18

Data presented as mean ± (SD), frequencies or percentages

^**1**^T Test

^2^Fisher exact test

*Without Penicillin/Streptococcus (-)

**Without Penicillin/Streptococcus (+)

***With Penicillin/Streptococcus (-)

****With Penicillin/Streptococcus (+)

### 16S rRNA based microbiota analysis

After quality filtering, a total of 575,539 high quality sequences were retained with an average of 4,031 sequences/sample (mean of 200 bases in length and maximum expected error of 0.5). Good’s coverage was between 84% and 100% sequencing coverage at 97% similarity cutoff, indicating that the numbers of sequences were sufficient for the communities measured in all libraries.

**Fig 1:**
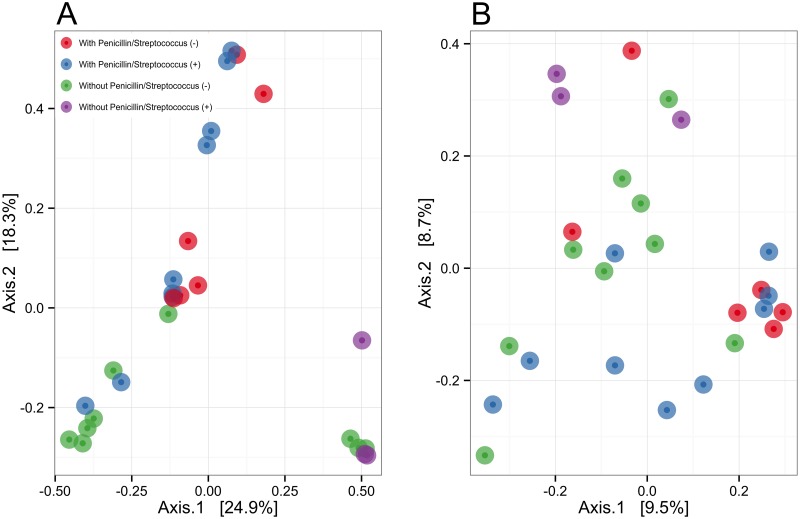
A and B. Principal coordinates plot (PCoA) representing clusters of microbial communities from mother’s vagina. (A) Bray Curtis distance metrics, which accounts for differences in the relative abundance of taxonomic unities. (B) Binary distance metrics, which accounts for presence/absence of taxonomic unities. Each point represents the microbial community of an individual sample with colors indicating treatments.

PCoA was applied to compare vaginal microbial communities among treatments using Bray Curtis and Binary distances at 97% cutoff similarity level for OTU grouping (Fig 1A and 1B). The Bray Curtis metrics showed distinctive groups of vaginal microbial communities associated with treatments. In addition, multivariate ANOVA based on Bray Curtis dissimilarities (Adonis) confirmed the differences among treatments (R^2^ = 0.23; *p* = 0.002). The R^2^ value indicated that approximately 23% of the variation in distances is explained by treatments. The Binary metrics also showed grouping of samples by both PCoA analysis (Fig 1B) and multivariate ANOVA based on Binary dissimilarities (R^2^ = 0.13 *p* = 0.03). Nevertheless, the low R^2^ value suggests that the differences in the relative abundance of taxonomic unities between treatments were more important than the differences in the presence/absence of them. Suspected confounding variables that were collected with our questionnaires (maternal comorbidities) were tested by multivariate ANOVA S1 Table. Besides antibiotic usage, none of the variables tested shown significant effect on microbial communities at p-value < 0.05.

**Fig 2:**
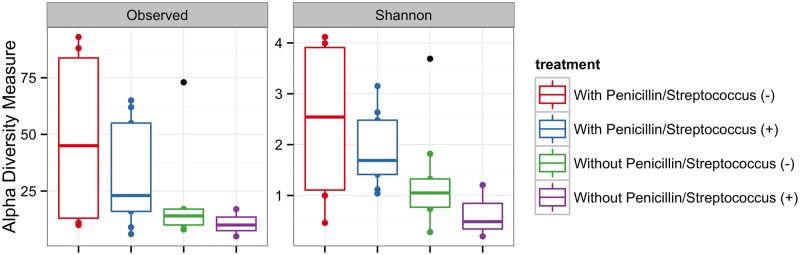
Alpha diversity measurements of vaginal microbial communities from pregnant women under different treatments. The boxes spans the first quartile to the third quartile, the horizontal line inside the boxes represents the median. Lines extending vertically from the boxes indicate variability outside the upper and lower quartiles and the single black circles indicate outliers. After detecting overall differences in beta and alpha diversity among samples, an analysis of the specific taxonomic composition of mothers’ vagina was performed in order to identify the microbes responsible for differences among treatments. Significant differences (*p*< 0.05) were observed in the abundance of *Lactobacillus* (Fig 3). *Lactobacillus* spp. was the most abundant genus found in the swab samples from the group of pregnant women without use of penicillin contributing 88.1% and 68.5% with positive and negative *Streptococcus* screening, respectively. In contrast, *Lactobacillus* contributed only 13.1% and 6.0% of the total vaginal microbial community found in the cases with use of penicillin with positive and negative screening for GBS, respectively. *Pseudomonas* was the second most abundant genus among samples contributing up to 6.0% of the total community in those women without use of penicillin. On the other hand, *Pseudomonas* contributed up to 17.5% of the total community in the cases with use of penicillin. Although the average abundance of *Pseudomonas* suggested differences between treatments, such difference was not significant (*p* = 0.134).

To investigate differences in bacterial diversity between treatments we observed OTU richness and calculated Shannon diversity index (Fig 2). According to these alpha diversity measurements, the group with penicillin presented a significantly higher microbial diversity. The mean observed richness within the microbial community from mothers with use of penicillin and negative *Streptococcus* screening was 47.6 and the Shannon diversity index was 2.42 whereas the observed microbial richness from mothers that received penicillin and were positive for *Streptococcus* screening was 30.8 and the Shannon diversity index was 1.86. On the other hand, the mean observed richness within the microbial community from mothers without use of penicillin and negative *Streptococcus* screening was 18.3 and the Shannon diversity index was 1.28. The mean observed richness within the microbial community from mothers without use of penicillin and positive *Streptococcus* screening was 10 and the Shannon diversity index was 0.65. Altogether these alpha diversity measurements indicate greater richness and evenness of the community in mothers with penicillin and lower richness and evenness in mothers with *Streptococcus* infection.

**Fig 3:**
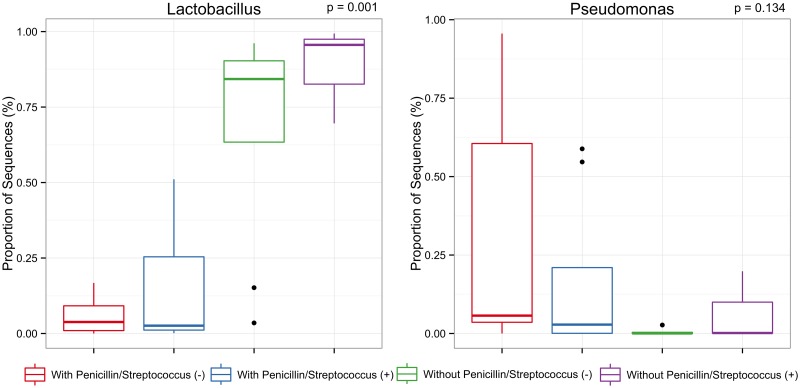
Differentially abundant taxa found in vaginal samples of pregnant women whose abundance differed statistically between treatments. Comparisons were performed using ANOVA for multiple comparisons followed by the Tukey-Kramer *post hoc* test.

**Fig 4:**
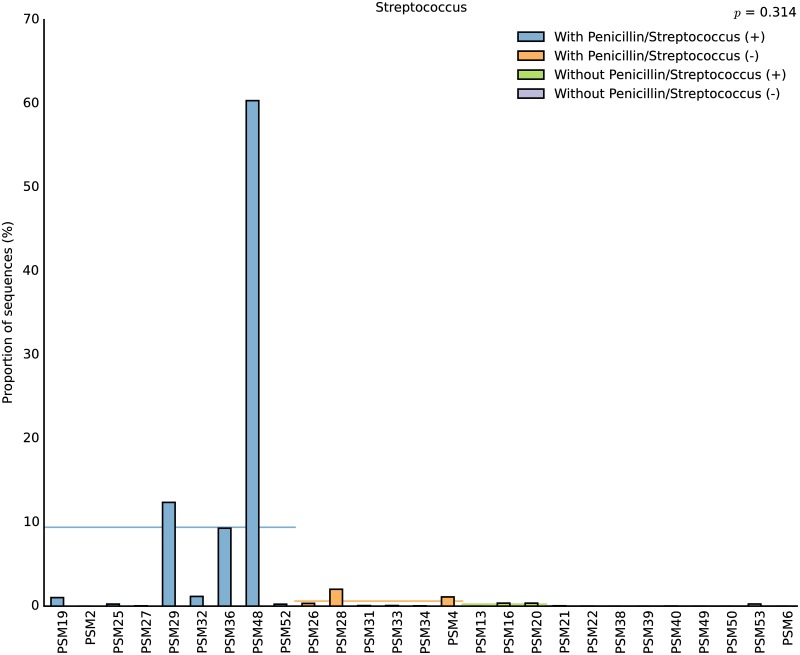
Relative abundance of *Streptococcus* within each patient. Horizontal lines represent the average proportion of sequences per treatment.

In addition to the clinical screening, the abundance of *Streptococcus* spp. was also investigated by our sequencing approach. *Streptococcus* was detected in 74% of our samples irrespective of treatment (Fig 4). The relative abundance of this genus varied among cases with positive prenatal culture screening reaching up to 60% of the total microbial community in cases with severe infection or below 0.35% in less severe cases. *Streptococcus* was also detected in 44% of cases with negative culture screening but in a very low abundance (below 0.2%). These results indicate that *Streptococcus* is naturally present in the healthy pregnant woman although in very low abundance.

## Discussion

In this study, we collected vaginal swab samples from pregnant women under four different conditions associated with antibiotic prophylaxis and presence of positive culture screening for GBS. DNA extraction, PCR amplification, primer choice, and sequencing platform all have the potential to affect results. While these factors can lead to biases we used established protocols that minimize concerns of comparing our results with those of other studies. We combined a highly robust data generation methodology, with a stringent bioinformatics approach, thus limiting the impact of any sequencing errors [12,13]. Our bioinformatics approach and experimental design was aimed at identifying which microorganisms are most important in maintaining the structure and interactions of microbial communities in healthy pregnant women and better understand the main modifications caused by penicillin usage in the vagina. Resolving microbial species based on 16S rRNA gene is difficult and there is currently no consensus on an approach to define species purely based on 16S rRNA gene sequence data [19]. In this regard, the analysis and interpretation of our dataset must be viewed with caution since many microbial genera, contain both pathogenic and commensal species. We used appropriated statistical methods to deal with small sample sizes, however, caution is warranted in making inferences on antibiotic usage on vaginal microbiome community shifts since the approach is sufficiently powered only to show big differences.

Although culture-independent studies comparing the human microbial communities with and without antibiotic disturbance have been carried out [20,21], this study is the first to have done so with vaginal samples of pregnant women with and without intrapartum antibiotic. The vaginal sites (introitus, midpoint and posterior fornix) harbor particularly simple microbial communities with lowest alpha diversity at the genus level compared to other body habitats like oral, skin and gut [22]. On the other hand, at the Operational Taxonomy Unities the alpha diversity can be considered high due to the presence of distinct *Lactobacillus* spp. [22]. Thus, it appears that the vagina of healthy pregnant women is predominantly colonized by *Lactobacillus* spp. while the vagina of pregnant women under intrapartum antibiotic prophylaxis and with positive screening for GBS presented significant differences in microbial composition and diversity. Several studies have examined the vaginal microbiota during pregnancy using cultivation-independent techniques [6–8,23–25]. Collectively, these studies found the vaginal communities of pregnant women to be dominated by *Lactobacillus* species and characterized by lower richness, diversity and high stability than in non-pregnant women. Indeed, high Nugent scores, used to diagnose bacterial vaginosis, were associated with *Aerococcus*, *Anaeroglobus*, *Anaerotruncus*, *Atopobium*, *Coriobacteriaceae*, *Dialister*, *Eggerthella*, *Gardnerella*, *Gemella*, *Megasphaera*, *Mobiluncus*, *Parvimonas*, *Peptoiphilus*, *Prevotella*, *Porphyomonas*, *Prevotellaceae*, *Ruminococcaceae*, and *Snethia* in a cohort of 396 North American women made up by Asian, white, black and Hispanic ethnic backgrounds [5]. The same study also correlated low Nugent scores with the presence of *Lactobacillus* indicating that the presence of this genus is positively correlated with a healthy vaginal environment. The *Lactobacillus* genus is a lactic acid producing microbial group, which can low the vaginal pH (4 ± 0.5) creating an environmental barrier against pathogen invasion [26,27]. After a systematic review of 63 studies that used at least one molecular technique to characterize the vaginal microbiome, van de Wijgert et al. [28] concluded that the vaginal microbiome dominated by *Lactobacillus* spp. are associated with healthy vaginal environment and that bacterial vaginosis is best described as a polybacterial dysbiosis. As discussed by MacLntyre et al. [29] such *Lactobacillus* spp. dominated environment is likely shaped by the increase in the concentration of oestrogen during pregnancy, which in turns, drive the maturation of the vaginal epithelium leading to the accumulation of glycogen. The glycogen is braked down by the vaginal alpha-amylase into maltose, maltotriose, and maltotetraose that support *Lactobacillus* spp. colonization. This is in line with the findings of Li and Ma [30] who tested the biodiversity assembly theory using human microbiome datasets and concluded that in most cases, it is the host environment that ultimately shapes the community assembly.

As the pregnancy progressed no dramatic changes in the taxonomic structure of the vaginal microbial communities had been detected [31] however, changes in the relative abundance of four *Lactobacillus* spp. (*L*. *crispatus*, *L*. *jensenii*, *L*. *gasseri* and *L*. *vaginalis*) as a function of gestational age were already observed [24].

Antibiotics may induce alterations in the commensal microbiota of the birth canal in pregnant women causing rapid and profound shifts in the vaginal microbiota following antibiotic treatment for bacterial vaginosis on a scale of hours [32]. Stokholm *et al*. [33] studied the effect of oral antibiotic administration during pregnancy at 36 gestational weeks on commensal vaginal bacterial colonization. They found that women treated with antibiotics in the third trimester of pregnancy were more often colonized by *Escherichia coli* than women without antibiotic treatment in the third trimester. Also, according to the authors, antibiotic administration did not significantly influence the vaginal GBS or *S*. *aureus* colonization rates.

In this work only mothers delivering at <32 weeks gestational age were analyzed so our results might be limited to understand the changes due to antibiotic usage only in the vaginal ecosystem of pregnant women who subsequently have preterm delivery. At this moment we studied just mothers of very preterm newborns because we are interested in the repercussion of maternal antibiotic therapy on the preterm microbiota. However the data might also be meaningful in assessing the antibiotic effect on who delivered at term if we consider that no differences in relative abundance of bacterial phylotypes between women who delivered at term or had spontaneous preterm delivery were detected [24].

It is important to note that having found *Streptococcus* in greater abundance in penicillin treated subjects does not necessarily means that the use of antibiotic increased the abundance of this genus. No samples were collected before the antibiotic administration therefore the initial abundance of *Streptococcus* was unknown. The use of penicillin might have reduced the abundance of *Streptococcus* or penicillin just fail to eradicate GBS as reported before [34,35]. Our experiment design allowed us to detect increase of diversity due to use of penicillin. Antibiotic perturbation was previously associated with loss of microbial diversity and shifts in community composition [21], and according to our results severe cases of GBS infection can also decrease vaginal diversity due to dominance of *Streptococcus*. The antibiotic usage can eliminate more abundant microbes allowing for a more even distribution of microbial species that in turn allow for sampling more OTUs with the same sequencing effort. In this sense we might not observe a real increase of diversity but a strong reduction of dominant microbial species. When studying microbial communities from the English Chanel using deep sequencing of the 16S rRNA and comparing against shallow sequenced time points, Caporaso *et al*. [36] also noticed the effect of shallow sequencing in microbial diversity measurements. The deep sequenced time point maintained 95.4% of the combined data acquired by shallow sequencing during 6 years. The data suggested that the majority of taxa are always present but just in different proportions. Whether the antibiotic reduced diversity or reduced more abundant microbes allowing a more even microbial sampling is unknown however the disturbing effect of antibiotic cannot be neglected.

We were not able to establish a relationship between modification of maternal vaginal microbiota and early-onset neonatal sepsis. We studied 27 women and the prevalence of early-onset neonatal sepsis was none. According to *Stoll et al*. [35] the prevalence of neonatal sepsis might be around 1.9%.

In conclusion, our study showed, with a high resolution technique that vaginal communities of healthy pregnant women was dominated by *Lactobacillu*s species and present low genus diversity, while *Streptococcus* colonized women under peripartum penicillin presented a modified vaginal microbiota composition with low abundance of *Lactobacillus* and altered microbial composition.

## Supporting information

S1 Table Multivariate analysis of variance based on microbial communities dissimilarity matrix showing the differences among suspecting confounding variables. Df = degrees of freedom; SS = sum of squares; MS = mean sum of squares.
(DOC)
