# Supplementary material for: Diversity and composition of vaginal microbiota of pregnant women at risk for transmitting Group B Streptococcus treated with intrapartum penicillin
Source: PLoS One. 2017 Feb 8;12(2):e0169916. doi: 10.1371/journal.pone.0169916 (PMC5298327; doi:10.1371/journal.pone.0169916)
Supplement: S1 Table — Df = degrees of freedom; SS = sum of squares; MS = mean sum of squares. (DOC) [file pone.0169916.s001.doc]

Supplementary Table S1. Multivariate analysis of variance based on microbial communities dissimilarity matrix showing the differences among suspecting confounding variables.

|  | Df | SS | MS | F.Model | R2 | Pr(>F) |
| --- | --- | --- | --- | --- | --- | --- |
| Maternal_age | 1 | 0.4183 | 0.41827 | 1.01234 | 0.03946 | 0.418 |
| Gestational_age | 1 | 0.2774 | 0.27739 | 0.67138 | 0.02617 | 0.777 |
| Chorioamnionitis | 1 | 0.4598 | 0.45984 | 1.11296 | 0.04339 | 0.355 |
| Preeclampsia | 1 | 0.3423 | 0.34231 | 0.8285 | 0.0323 | 0.593 |
| Gestational_diabetes | 1 | 0.508 | 0.50799 | 1.22951 | 0.04793 | 0.264 |
| Maternal_age:Gestational_age | 1 | 0.4419 | 0.44193 | 1.06963 | 0.0417 | 0.366 |
| Maternal_age:Chorioamnionitis | 1 | 0.4102 | 0.41018 | 0.99278 | 0.0387 | 0.425 |
| Gestational_age:Chorioamnionitis | 1 | 0.2313 | 0.2313 | 0.55982 | 0.02182 | 0.902 |
| Maternal_age:Preeclampsia | 1 | 0.3683 | 0.36835 | 0.89153 | 0.03475 | 0.534 |
| Gestational_age:Preeclampsia | 1 | 0.4517 | 0.45168 | 1.09321 | 0.04262 | 0.379 |
| Chorioamnionitis:Preeclampsia | 1 | 0.1828 | 0.1828 | 0.44244 | 0.01725 | 0.955 |
| Maternal_age:Gestational_diabetes | 1 | 0.4484 | 0.44835 | 1.08516 | 0.0423 | 0.379 |
| Gestational_age:Gestational_diabetes | 1 | 0.5339 | 0.53393 | 1.29229 | 0.05038 | 0.241 |
| Chorioamnionitis:Gestational_diabetes | 1 | 0.3832 | 0.38325 | 0.92759 | 0.03616 | 0.505 |
| Preeclampsia:Gestational_diabetes | 1 | 0.4251 | 0.42506 | 1.02879 | 0.0401 | 0.577 |
| Maternal_age:Gestational_age:Chorioamnionitis | 1 | 0.5162 | 0.51619 | 1.24936 | 0.0487 | 0.256 |
| Maternal_age:Gestational_age:Preeclampsia | 1 | 0.4815 | 0.48154 | 1.16549 | 0.04543 | 0.312 |
| Residuals | 9 | 3.7185 | 0.41317 |  | 0.35084 |  |
| Total | 26 | 10.5988 |  |  | 1 |  |

Df = degrees of freedom; SS = sum of squares; MS = mean sum of squares
